# A randomized controlled trial to assess the clinical and cost effectiveness of a nurse-led Antenatal Asthma Management Service in South Australia (AAMS study)

**DOI:** 10.1186/1471-2393-14-9

**Published:** 2014-01-08

**Authors:** Luke E Grzeskowiak, Gustaaf Dekker, Karen Rivers, Kate Roberts-Thomson, Anil Roy, Brian Smith, Jeffery Bowden, Robert Bryce, Michael Davies, Justin Beilby, Anne Wilson, Philippa Middleton, Richard Ruffin, Jonathan Karnon, Vicki L Clifton

**Affiliations:** 1 The Robinson Institute, The University of Adelaide, Haydown Road, 5112 Adelaide, SA, Australia; 2 Department of Respiratory Medicine, Lyell McEwin Hospital, Adelaide, SA, Australia; 3 Department of Obstetrics and Gynaecology, Flinders Medical Centre, Adelaide, SA, Australia; 4 Faculty of Health Sciences, The University of Adelaide, Adelaide, SA, Australia; 5 School of Medicine, Flinders University, Adelaide, SA, Australia; 6 School of Medicine, University of New South Wales, NSW, Australia; 7 Department of Medicine, The University of Adelaide, Adelaide, Australia; 8 School of Population Health and Clinical Practice, The University of Adelaide, Adelaide, SA, Australia

**Keywords:** Asthma, Pregnancy, Inhaled corticosteroids, Randomized controlled trial, Antenatal care, Intervention

## Abstract

**Background:**

Pregnancy presents a unique situation for the management of asthma as it can alter the course of asthma severity and its treatment, which in turn can affect pregnancy outcomes. Despite awareness of the substantial adverse effects associated with asthma during pregnancy, little has been done to improve its management and reduce associated perinatal morbidity and mortality. The aim of this randomized controlled trial is to evaluate the clinical and cost effectiveness of an Antenatal Asthma Management Service.

**Methods/design:**

*Design:* Multicentre, randomized controlled trial.

*Inclusion criteria:* Women with physician diagnosed asthma, which is not currently in remission, who are less than 20 weeks gestation with a singleton pregnancy and do not have a chronic medical condition.

*Trial entry and randomization:* Eligible women with asthma, stratified by treatment site, disease severity and parity, will be randomized into either the ‘Standard Care Group’ or the ‘Intervention Group’.

*Study groups:* Both groups will be followed prospectively throughout pregnancy. Women in the ‘Standard Care Group’ will receive routine obstetric care reflecting current clinical practice in Australian hospitals. Women in the ‘Intervention Group’ will receive additional care through the nurse-led Antenatal Asthma Management Service, based in the antenatal outpatient clinic. Women will receive asthma education with a full assessment of their asthma at 18, 24, 30 and 36 weeks gestation. Each antenatal visit will include a 60 min session where asthma management skills are assessed including: medication adherence and knowledge, inhaler device technique, recognition of asthma deterioration and possession of a written asthma action plan. Furthermore, subjects will receive education about asthma control and management skills including trigger avoidance and smoking cessation counseling when appropriate.

*Primary study outcome:* Asthma exacerbations during pregnancy.

*Sample size:* A sample size of 378 women will be sufficient to show an absolute reduction in asthma exacerbations during pregnancy of 20% (alpha 0.05 two-tailed, 90% power, 5% loss to follow-up).

**Discussion:**

The integration of an asthma education program within the antenatal clinic setting has the significant potential to improve the participation of pregnant women in the self-management of their asthma, reduce asthma exacerbations and improve perinatal health outcomes.

**Trial registration:**

ACTRN12613000244707

## Background

Pregnancy presents a unique situation for the management of asthma as it can alter the course of asthma severity and its treatment, which in turn can affect pregnancy outcomes. Despite awareness of the substantial adverse effects associated with asthma during pregnancy, little has been done to improve its management and reduce associated perinatal morbidity and mortality. Asthma is the most prevalent complication to affect human pregnancy in Australia, affecting an estimated 12% of pregnant women, or 36,000 pregnancies each year [1]. The significance of asthma in pregnancy is highlighted in a recent meta-analysis, demonstrating clear associations between asthma in pregnancy and adverse perinatal outcomes, including increased risk of low birth weight (RR 1.46; 1.22-1.75), small-for-gestational age (RR 1.22; 1.14-1.31), preterm delivery (RR 1.41; 1.22-1.61) and pre-eclampsia (RR 1.54; 1.32-1.81) [2]. The adverse effects of asthma in pregnancy are related to asthma severity and the intensity of treatment [3-5]. Studies of women with well controlled asthma, which involves the regular use of inhaled corticosteroids (ICS) to inhibit inflammatory mechanisms at a systemic level, seldom demonstrate adverse effects on fetal outcome [3,6]. In contrast, regular use of oral corticosteroids (OCS), used in the management of acute asthma exacerbations and poorly controlled asthma during pregnancy, has been associated with an increased incidence of low birth weight babies and preterm delivery [7]. A number of studies have examined the effects of maternal asthma during pregnancy, in the presence and absence of ICS treatment, on placental function and fetal development. These studies have demonstrated that use of ICS for the treatment of asthma did not affect fetal growth and that maternal asthma without treatment had a greater impact on the fetus and placenta [8]. Furthermore, acute asthma exacerbations were identified as the most significant event to affect fetal morbidity and mortality in pregnancies complicated by asthma [9,10]. That is, poor asthma control is a greater risk factor for an adverse outcome during pregnancy than ICS use [10]. In contrast, evidence demonstrates little or no increased risk of adverse maternal or fetal complications in situations where asthma is well controlled throughout pregnancy [6,11]. These findings point to the fact that optimization of asthma management and asthma control is key to improving perinatal outcomes. As such, pregnancy represents a significant indication to optimize therapy and maximize lung function in order to reduce the risk of acute exacerbation and resultant adverse health outcomes. This study will address these issues through the development and evaluation of cost effective ways of improving antenatal management of asthma.

### Current care of pregnant women with asthma

Clear guidelines exist for the management of asthma during pregnancy, which involves managing asthma medications in pregnant women in the same manner as a non-pregnant adult [12]. This relates to assessing asthma control and adjusting inhaled medications accordingly at regular intervals during pregnancy. The significant burden associated with asthma is highlighted through National Health Surveys which identified that approximately 12% of women of childbearing age report having current asthma [1,13]. While no studies have directly assessed the prevalence of asthma amongst pregnant women in Australia, prospective cohort studies [14] and antenatal surveys [15] undertaken amongst pregnant women have identified a similar prevalence of approximately 12-13%. Despite this estimated high prevalence, only 6% of pregnancies are reported to be affected by asthma in Australian perinatal statistics collections [16]. This apparent under reporting of asthma prevalence could be a result of the fact that asthma is not well recognized as a significant co-morbidity of pregnancy and has the potential to result in reduced capacity to manage the disease during pregnancy.

Currently, there is no structured approach in which asthma in pregnancy is managed. While a small number of women with very severe asthma receive monitoring during pregnancy in the antenatal clinic with respiratory physician support, treatment is often limited to women self-managing their asthma with support from their family doctor. Current asthma management appears to be focused on a reactive, rather than proactive, approach, which is demonstrated by the fact that only 15% of pregnant women with asthma involved in a prospective study had an asthma action plan [17]. Therefore, there is clear evidence that current care of pregnant asthmatic women could be significantly improved and there is an immediate need for evidence based approaches for improving clinical practice.

### Asthma exacerbations during pregnancy are associated with an adverse outcome

Exacerbations are a key feature of asthma and ideal target for improving asthma control and related outcomes. The definition of an asthma exacerbation is any asthma related event that involved one or more of the following; a hospital admission, an unscheduled doctor visit, a course of oral steroids or an increase in medication use. A systematic review identified that 6% of asthmatic women are hospitalized for a severe exacerbation during pregnancy, and that 20% have a severe exacerbation during pregnancy requiring medical intervention [7]. Furthermore, evidence demonstrates that the rates of exacerbations during pregnancy increases according to asthma severity [18]. Among those with mild, moderate and severe asthma the exacerbation rates have been reported as 13%, 26% and 52%, respectively [18]. The corresponding rates of exacerbations leading to hospitalization were 2%, 7% and 27% [18]. Relying solely on asthma severity, however, can be misleading as asthma can worsen during pregnancy regardless of maternal pre-pregnancy severity [7,10]. That is to say; an asthmatic woman can be a mild asthmatic pre-pregnancy and become a severe asthmatic during pregnancy. In addition, the severity of an exacerbation does not always reflect underlying asthma severity (i.e. women with mild asthma can have a severe exacerbation). This means it is difficult to identify accurately those women at risk of worsening asthma during pregnancy.

### Risk factors for exacerbations during pregnancy

While exacerbations can occur at any time during pregnancy, evidence from a prospective cohort of women with asthma identified that exacerbations were most likely in the second and third trimesters between weeks 17 and 34, with a peak incidence around 25 weeks gestation [10]. Risk factors associated with asthma exacerbations during pregnancy include respiratory viral infections and a lack of appropriate treatment with inhaled corticosteroids (ICS). Asthmatics appear to be more susceptible to respiratory tract infection during pregnancy and also have an increased risk of community-acquired pneumonia [19]. This suggests that the combination of asthma and pregnancy may significantly increase the risk of infection and may result in acute asthma exacerbations during pregnancy. Improvements in asthma management that address these issues and reduce exacerbation rates on a population basis are needed.

### Treatment of asthma and asthma exacerbations during pregnancy

Pregnancy has a unique effect on decisions around asthma control and management. Despite guidelines recommending the continuing of usual asthma medications during pregnancy, it is not uncommon for women to cease their medications, with or without consultation with their doctors [20]. These decisions may be driven by a perceived lack of need to continue their medications, a lack of support and guidance from health professionals regarding how to manage their asthma medications and/or concerns regarding the safety of asthma medications during pregnancy [21]. These concerns may not be unique to women themselves, as a previous survey demonstrated that in situations where asthma was well controlled, over 25% of family physicians would instruct their pregnant patients to decrease or discontinue asthma medication during pregnancy, despite the potential to negatively impact on subsequent asthma control [22]. Furthermore, a study conducted in the US found that pregnant women with an asthma exacerbation were significantly less likely to be given oral steroids either in the emergency department or on discharge from hospital than non-pregnant asthmatic women (50.8% vs. 72.4%) [23]. Subsequently, pregnant asthmatic women were three times more likely to report an on-going asthma exacerbation following discharge when compared with non-pregnant asthmatic women [23]. The lack of treatment of an exacerbation during pregnancy can severely compromise the fetus by reducing the oxygen supply to the utero-placental unit and has a more significant impact on the fetus than the medication itself [24]. Daily ICS use has previously been identified as a safe medical approach to controlling asthma during pregnancy and reducing the risk of exacerbation [24-27]. The uncertainty, concerns and variability in practice surrounding medication use and asthma control during pregnancy emphasizes the key need for a dedicated Antenatal Asthma Management Service.

### Existing evidence on the provision of asthma self-management skills and education during pregnancy

Previous research undertaken by the research team has demonstrated that asthma education combined with an asthma action plan administered in the antenatal clinic by a respiratory nurse is associated with a significant improvement in birth weight relative to women who did not use an action plan during pregnancy [17], and may reduce the risk of exacerbations as a result of improved asthma control.

In this study asthmatic women were prospectively recruited at their first antenatal visit and attended a nurse-led asthma management service at the antenatal clinic at 20 and 33 weeks gestation (n = 211) [17]. Lung function, asthma symptoms, compliance with medications, medication knowledge and inhaler technique were assessed. Women were given a written asthma action plan and educated in peak flow monitoring for home use. At the first visit, the majority of women were identified as having poor asthma self management skills and knowledge, regardless of the severity of their disease [17]. Limitations of this study were that the service was not assessed as part of a RCT (i.e. all women received intervention) and small sample size, limiting the ability to effectively assess the clinical and cost effectiveness of the intervention.

As a follow-up to this study a RCT was undertaken to investigate the clinical use of exhaled fraction of nitric oxide (F_E_NO), as a measure of inflammation, to adjust asthma management during pregnancy. Use of F_E_NO to adjust asthma treatment was associated with a significant reduction in the exacerbation rate during pregnancy (0.288 vs. 0.615; p = 0.001) and a reduction in neonatal hospitalizations (8% vs. 17%; p = 0.046) [28]. The major limitation of this trial, however, was that only non-smokers were enrolled in the study as cigarette use inhibits exhaled NO production in asthmatics and therefore can significantly mask the interpretation of NO data [29]. This significantly restricts the generalisability of this approach given the high proportion of asthmatic women who also smoke during pregnancy (reported to be at least 34% in one Australian cohort study) [14]. The applicability of F_E_NO to routine clinical settings is further limited due to issues around expense and accessibility. In addition, as all women in the study attended a respiratory nurse-led asthma management service, this limits the ability to adequately assess the clinical and cost effectiveness of this intervention.

While these two studies demonstrate the feasibility of providing an Asthma Management Service in the antenatal setting and the potential significant benefits it may achieve, an RCT is the only rigorous approach to truly determine the clinical and cost effectiveness of a nurse-led Antenatal Asthma Management Service versus standard clinical care.

### Summary

Given the scarcity of available health resources, the evaluation of the clinical and cost effectiveness of this intervention is important to establish whether our previous research findings can be translated into an effective clinical practice integrated with routine antenatal services. The proposed model makes use of specially trained respiratory nurses to deliver an Antenatal Asthma Management Service. The use of nurse-led clinics can facilitate specialist intervention at times of identified patient need, while nursing health practice allows for the introduction of preventative health practices to patients which cannot always be introduced by doctors due to limitations on time. The integration of an adapted model of an asthma education program within the antenatal clinic setting has the significant potential to improve the participation rate of pregnant women in the self-management of their asthma, reduce asthma exacerbations and thus improve perinatal health outcomes. It will also integrate well with a shared care model where asthma education occurs as part of the antenatal visits to the hospital with communication with the primary care physician and specialist physician. Positive outcomes will demonstrate the potential value of nurse led interventions, leading to closer consideration of other clinical conditions in which similar nurse-led initiatives could be evaluated and implemented.

### Aims and objectives of this trial

The aim of this randomized controlled trial is to evaluate the clinical and cost effectiveness of an Antenatal Asthma Management Service.

### Hypothesis

The provision of a nurse-led Antenatal Asthma Management Service (AAMS) is a cost effective intervention to improve asthma management and maternal health and well being and reduce the incidence of asthma exacerbations.

## Methods/design

### Ethics statement

Ethics approval was granted by the Human Research Ethics Committee of The Queen Elizabeth Hospital, Lyell McEwin Hospital and Modbury Hospital in Adelaide, South Australia (HREC/12/TQEHLMH/73).

### Study design

Multicentre parallel-group randomized controlled trial.

### Setting

This study will involve two public hospital antenatal clinics which account for 40% of all births in South Australia, Australia. These hospitals are the Lyell McEwin Hospital (LMH) and Flinders Medical Centre (FMC). A clinical study has been running at the LMH throughout 2012 to demonstrate the feasibility of implementing an Antenatal Asthma Management Service. During this period approximately 40 referrals have been received each month from the antenatal outpatient clinic for women with asthma. This represents an estimated prevalence of asthma in this population of 14%. This high prevalence is likely due to the fact that more than 70% of women with asthma use public health care and that the prevalence of asthma is higher among those with lower socioeconomic status [14], reflective of the area in which LMH is situated. In comparison to the LMH, FMC is situated in a region of higher SES than LMH (SA Health Atlas). This will enable the clinical and cost-effectiveness of the service to be investigated in two diverse settings, as previous research has identified that SES is a significant risk factor for outcomes related to maternal asthma [30].

### Recruitment

All pregnant women with asthma attending their first booking visit (approximately 12 weeks gestation) at the antenatal outpatient clinic will be identified by the attending midwife. The midwife will ask several questions to establish the presence of asthma including “have you been told by a doctor that you have asthma? As well as “Have you used any asthma medications in the last year such as salbutamol or a preventer?” Women identified as having asthma will be referred by the attending midwife and will be subsequently interviewed by a research midwife associated with this study who will explain the project to them by providing both written and verbal information.

To be eligible, participants must be less than 20 weeks gestation at the time of recruitment, have asthma which has been previously diagnosed by a doctor and is not currently in remission (remission defined as no asthma symptoms and no use of asthma medications in previous year), aged 18–45 years, expected to give birth to a singleton and be able to speak English. Participants will be excluded from the study if they have previously participated in an Asthma study run at the hospital or have a pre-existing chronic medical condition (i.e. diabetes, hypertension, cardiac disease, HIV/hepatitis, renal disease, haematology disorder (i.e. thalassaemia, thrombophilia), thyroid disorder, psychiatric disease requiring therapy with antidepressant or antipsychotic, epilepsy).

### Data collection at trial entry

Eligible women who agree to take part in the study and sign an informed consent, will be asked to complete a baseline assessment of their asthma. Participants will be asked routine questions to determine their asthma severity in accordance with the National Asthma Council Asthma Management Handbook classifications [12]. This classification takes into account asthma medications, symptoms and spirometry results to reach an approximate baseline asthma severity classification in women with **treated** asthma. Women will be classified as intermittent, mild, moderate or severe. In addition, their asthma control will be assessed by administering the Juniper Asthma Control Questionnaire (ACQ) (how many days in the past week the subject has been affected by night time symptoms, morning symptoms, activity limitation due to asthma as well as frequency of β2-agonist use) [17]. Forced expiratory volume at one second (FEV1) and forced vital capacity (FVC) will be measured by an Easy One spirometer before and after administration of salbutamol in all subjects. FEV1% predicted will be calculated based on the patients age, ethnicity and height using the equations of Gore *et al.*[31]. Further data will be collected using a cold and flu questionnaire to determine the impact of illness on current asthma control. These validated questionnaires, in addition to a structured data collection form, will be used to determine current asthma therapy and control, current asthma triggers and co-morbidity and past history including frequency of oral corticosteroid use and previous hospital admissions for asthma. In addition, data will be collected on smoking (direct and passive exposure), Quality of Life (Mini Asthma Quality of Life Questionnaire [32]) and psychological variables using the Perceived Control of Asthma Questionnaire [33], Brief Illness Perception Questionnaire [34], Medical Adherence Report Scale-Asthma [35], Patient-Clinician Communication Questionnaire [36], Beliefs About Medicines Questionnaire [37], Teratogenic Risk Perception Questionnaire [38], and a purpose designed Asthma Knowledge Questionnaire. For all participants blood will be drawn for total IgE and RAST testing to assess atopy.

### Study groups and management

Eligible women with asthma will be randomized into one of two study groups: either the ‘Standard Care Group’ or the ‘Intervention Group’.

### Randomization

After the baseline assessment of asthma at 18 weeks gestation, women will be randomised to receive either standard care or nurse-led asthma management during pregnancy by contacting the central telephone randomization service at the University of Adelaide. The telephone randomization service will use a randomization schedule with balanced variable blocks, prepared by an investigator not involved with recruitment or clinical care. Stratification will be undertaken according to treatment site, parity and asthma severity. During the randomization call, eligibility will be checked and information collected to enable stratification and to assist in follow-up.

### Treatment schedules

#### Standard care group

Standard care will be as outlined in the SA Perinatal Practice Guideline for the treatment of asthma during pregnancy (Available from http://www.sahealth.sa.gov.au). This involves women with asthma self managing their disease during pregnancy and seeking guidance for asthma management when required from a midwife, obstetrician, respiratory physician or GP. Some women in this group will already be managed by a respiratory specialist and this will be continued. While women receiving standard care will be followed-up at exactly the same time points as those in the intervention group, no formal attentional control will be utilized. Notably, while some researchers advocate for the use of attention controls there is, at this point in time, a significant lack of understanding regarding what constitutes an appropriate attention control group. There is also a lack of understanding regarding the potential benefits (or biases) associated with the use of an attention control, limiting its ability to be utilized in this setting [39].

#### Intervention group

The intervention group will receive care through the nurse-led Antenatal Asthma Management Service, based in the antenatal outpatient clinic. The Antenatal Asthma Management Service will be led by a respiratory nurse with qualifications in asthma management and education and spirometry. The details of the methodology to be used for nurse-led respiratory care in our study have been previously published [17]. All asthmatic women will receive asthma education with a full assessment of their asthma at 18, 24, 30 and 36 weeks gestation conducted by a respiratory nurse. Each antenatal visit will include a 60 min session where asthma management skills are assessed including: medication adherence and knowledge, inhaler device technique, recognition of asthma deterioration and possession of a written asthma action plan [17]. In addition, subjects will receive education about asthma control and management skills including trigger avoidance and smoking cessation counseling when appropriate [17]. While it is standard antenatal care for all pregnant women to complete a smoking questionnaire at their first booking visit and be encouraged to quit smoking, an additional key component of the Antenatal Asthma Management Service will be in providing additional counseling on the importance of smoking cessation in relation to their asthma and the health of their baby. Women will be provided with information on the benefits of smoking cessation and direct referrals will be made to QuitSA for cessation support.

During the first visit at 18 weeks gestation, all subjects will be provided with an individualized written asthma action plan, which has been developed according to evidence based principles by the asthma educator using a standardized template following National Asthma Council recommendations [12]. In addition, current asthma management therapies will be evaluated and recommendations regarding optimal therapy will be made. If women are identified at their first antenatal visit as having unstable asthma, as determined by the Juniper Asthma Control Questionnaire (ACQ) score of >1.5 a recommendation will be made for step-up therapy in accordance with well-established clinical guidelines and previous studies [28]. If inhaled corticosteroids need to be prescribed an on-call respiratory physician will be contacted. A detailed asthma management report, including asthma action plan and recommended changes in therapy if required, will be forwarded to the participant’s preferred family physician (i.e. general practitioner) who will review each action plan and assess the appropriateness of recommended changes in therapy. This preferred provider will be the lead clinician responsible for her asthma management during pregnancy. All recommendations made through the Antenatal Asthma Management Service will be recorded and followed-up to determine their outcome (i.e. acceptance of recommendation).

During subsequent visits to the Antenatal Asthma Management Service, the respiratory nurse will continually review asthma control and management plans. Subjects assessed as having poor asthma control (ACQ > 1.5) or experiencing a current exacerbation will be referred to their usual family physician in the community or respiratory physician in the hospital for appropriate review. During antenatal appointments urgent medical review will be available for patients with an acute exacerbation. The respiratory nurse and designated family physician and/or respiratory physician will collaborate closely on appropriate step-up therapy for the participant.

Between visits, participants will be encouraged to follow their asthma action plan closely and see their family doctor if their asthma worsens. Those subjects who also attend specialist respiratory clinics will also have information forwarded to their physician for reference. Subjects will be encouraged to maintain these specialist visits. During the study, care received for asthma management from a GP, obstetrician or respiratory specialist will be noted.

**Figure 1:**
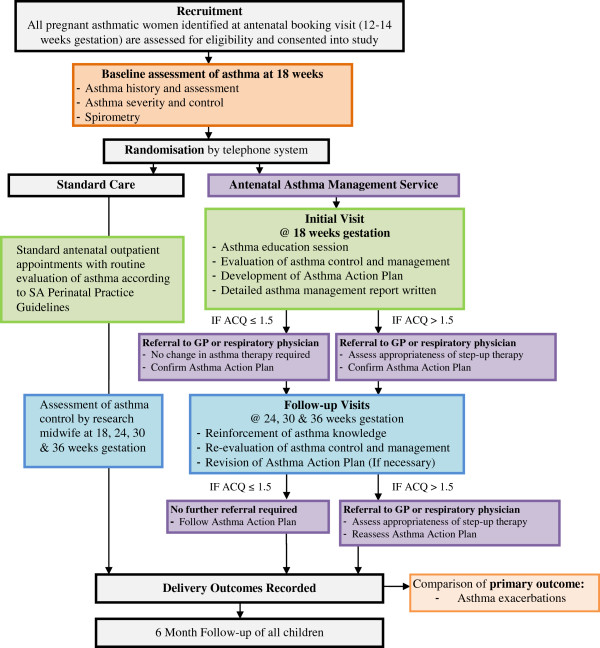
AAMS study design.

A flow chart of care is depicted in Figure 1.

### Data collection

All women will receive a baseline assessment of their asthma as previously described. Data will be collected on participants receiving standard care by a trained research midwife and those receiving the intervention by the respiratory nurse at 24, 30 and 36 weeks gestation. This will be undertaken using a structured data collection tool. At each time point asthma control will be assessed using the ACQ and spirometry. Further data will be collected using a cold and flu questionnaire to determine the impact of illness on current asthma control. A current pregnancy related history will be collected by research midwife interview for any unscheduled doctor visits due to asthma, an increased use of inhaled glucocorticoid, any visits to the Emergency department and any hospital admissions related to asthma, occurring since the previous visit. These data will be used to determine the number and timing of any asthma exacerbations occurring during the study. Data on asthma medication use will be utilized to calculate a cumulative ICS dose for each trimester. In addition, women will be asked to complete a questionnaire relating to smoking (direct and passive), and asthma related quality of life. Furthermore, data will be collected on the level of care received for asthma management from a GP, obstetrician or respiratory specialist (i.e. type and number of visits). Women receiving care through the Antental Asthma Management Service will have additional data collected on psychological variables as outlined in the section on data collection at trial entry (i.e. Perceived Control of Asthma Questionnaire [33]). Medicare Benefits Scheme (MBS) and Pharmaceutical Benefits Scheme (PBS) data relating to medical care and medication use throughout pregnancy will be accessed by participant consent. For all women, data on pregnancy complications, labour, delivery and birth outcomes will be abstracted from delivery case notes.

### Outcomes and measures

#### Primary study outcomes

The primary outcomes in this study will be the percentage of women that experienced exacerbations during pregnancy (moderate and severe) defined as events for which the participant sought medical attention (i.e. an unscheduled visit to a doctor, presentation to the emergency department room or admission to hospital, or when oral corticosteroids were used for treatment of asthma). Based on previous research undertaken by our research group it is hypothesised that participation in the Antenatal Asthma Management Service will result in a 20% reduction (absolute) in the incidence of asthma exacerbations during pregnancy in the intervention group compared to the control group [28].

#### Secondary study outcomes

For the mother, the secondary outcomes will be:

Asthma related variables defined as; ACQ score and FEV1 throughout gestation, asthma related quality of life, perceived control of asthma, medication adherence and asthma knowledge.

Adverse health outcomes defined as; hypertensive disorders of pregnancy, antepartum haemorrhage, gestational diabetes, preterm labour, need for antenatal hospitalization, weight gain during pregnancy, chorioamnionitis requiring antibiotics during labour, length of postnatal hospital stay, use of postnatal antiobitiocs and postpartum haemorrhage (≥500 mls) and emergency caesarean section.

For the infant/child, the secondary study outcomes will be:

Cases of infant morbidity defined as; Gestational age at delivery (determined by date of the last menstrual period and 18 week ultrasound), Preterm delivery (<37 weeks gestation), birth weight, birth length, head circumference IUGR (defined as those babies whose birth weight was less than or equal to the 5th centile), birth-weight-percentile (calculated using http://www.gestation.net), Apgar scores, congenital malformations, admission to neonatal intensive care unit or special care nursery and stillbirth.

### Sample size

In recognition of potential differences in the implementation and successfulness of the intervention across two distinct treatment sites, the sample size was calculated to ensure adequate statistical power within each treatment site. The total sample size of 378 pregnant women at baseline (189 in each group and each treatment site) was determined by power calculations (using 90% power and a 5% level of significance) using the primary outcomes of difference in exacerbation rate between treatment groups. We considered a 20% decrease (absolute reduction from 45% to 25%) in the incidence of asthma exacerbations as both achievable and clinically significant. The calculated sample size includes an estimated 15% loss to follow-up. We estimate there are at least 360 pregnancies complicated by asthma each year at each hospital and we will need to recruit 27% (n = 95 per site each year) of women presenting to the antenatal clinic per year to complete study recruitment over 2 years.

### Statistical analysis

Baseline characteristics of all randomized women will be compared descriptively between the study groups. Outcome comparisons will be made according to the treatment allocation at randomization on an ‘intention to treat’ basis. For the primary outcome, the difference in exacerbation rates between groups will be compared using a Poisson regression model. This will enable calculation of the incident rate ratio and associated 95% confidence interval.

For secondary outcomes, discrete outcomes (i.e. low birth weight) will be analysed using log binomial regression and continuous outcomes (i.e. birth weight, gestational age) analysed using linear regression. Continuous variables with repeated measurements (i.e. questionnaire results, ACQ score) will be analysed using a generalized linear mixed model (GLMM) with a random intercept for individuals to account for repeated measurements.

Planned sub-analyses will be undertaken to assess the effects of asthma control, FEV1, asthma medication use, cumulative ICS exposure, asthma knowledge and medication adherence, on the primary and secondary outcomes. SPSS Version 20 (SPSS Inc., Chicago, IL, USA). and Stata IC 11.1 (Stata, College Station, TX, USA) will be used for analyses. Statistical significance will be assessed at the 0.05 level using a two-sided comparative test.

### Cost effectiveness

A cost-effectiveness analysis will incorporate costs of providing standard care and the Antenatal Asthma Management Service, costs and quality of life effects associated with asthma-related adverse events, including mild and severe exacerbations, and fetal outcomes. This will determine whether, and under what circumstances, the provision of an Antenatal Asthma Management Service would be cost-effective. The economic evaluation will be performed from a societal and health care perspective. Direct medical costs (i.e. costs of intervention, asthma related consultations) as well as indirect costs (i.e. asthma related quality of life) will be taken into account. Relevant fixed unit costs associated with the delivery of standard care and the Antenatal Asthma Management Service intervention (i.e. costs of running the antenatal clinic, visits with respiratory nurse and respiratory specialist/preferred GP) will be estimated and allocated proportionally across the patients in the respective trial arms.

Additional costs associated with respiratory related health-care (i.e. asthma exacerbations, medication treatment costs), antenatal related health-care (i.e. pregnancy complications, medication treatment costs) and neonatal related health-care (i.e. admission to NICU) will be identified to determine a total cost estimate for the mother and child. We will obtain consent from patients to access all Medicare Benefits Schedule (MBS) (i.e. GP consultations) and Pharmaceutical Benefits Schedule (PBS) (i.e. medication costs) data to collate accurate data on unit costs associated with receiving either intervention during pregnancy. We will also obtain consent to access public hospital data, and so will capture emergency, outpatient, and inpatient encounters and the relevant unit costs associated with these encounters.

Missing cost data, for example, from patients who did not consent to access to their MBS, PBS, and public hospital data will be imputed using the Multiple Imputation using Chained Equations (MICE) approach.

To analyse long-term cost-effectiveness, extrapolation models for adverse neonatal outcomes (e.g. low birth weight and IUGR) will be developed, based on existing literature. A structured review of the literature will inform the costs and consequences (i.e. effects on Quality of Life) of adverse neonatal outcomes identified, which will be synthesised to model the short- and longer-term additional health-related events that a cohort of such babies would be expected to experience over their lifetime, or over a defined time horizon (e.g. 20 years). Similar lifetime extrapolation models have previously been developed in the areas of thalassaemia and sickle cell disease [40,41]. The resulting dataset will contain estimates of the short- and long-term costs and outcomes for all patients (mother and child), and will be analysed to estimate the expected costs and outcomes in the patient cohorts receiving the different levels of care.

To facilitate cost-effectiveness comparisons with interventions across other disease areas, outcomes for both mothers and children will be converted to quality adjusted life years (QALYs). Utility weights for mothers will be derived from responses to the AQoL [42], for which an Australian-based algorithm is available. The extrapolation models for fetal outcomes will incorporate literature-based health state utility weights for events included in the model to estimate QALYs gains in this population. The within trial analysis will report the costs and varied consequences (i.e. effects on mother and baby) of the different interventions. To inform comparisons with the cost-effectiveness of interventions across the health care system, the data from the study will be combined with the extrapolation models to estimate the expected incremental cost per QALY gained as a result of the Antenatal Asthma Management Service.

Every trial participant will have an estimated lifetime cost, and QALY value. If imbalances in baseline characteristics across the treatment groups are observed, propensity weighted generalized linear models will be used to generate adjusted mean cost and QALY estimates for each treatment group. In the absence of imbalance, the mean cost and QALY estimates in each group will inform mean incremental cost-effectiveness ratios, and incremental net monetary benefits.

Uncertainty will be represented using deterministic and probabilistic (bootstrapped) sensitivity analyses. The probabilistic analysis will inform credible intervals around the incremental cost-effectiveness ratios and cost-effectiveness acceptability curves based on alternative assumed monetary equivalent values of a QALY.

### Trial management

An independent data monitoring committee will be established, with terms of reference jointly defined between trial investigators and the independent committee members prior to study commencement. A multidisciplinary adverse events committee blinded to treatment allocation will review the cause of death for all maternal and infant deaths. These data will be made available to the independent data monitoring committee. In addition, the committee will conduct an interim analysis midway through the trial after the inclusion of 189 women. They will be unaware of treatment allocation when they judge data on effectiveness and safety and will compare these against the stopping rules outlined in the terms of reference. All study investigators have agreed to meet on a monthly basis to monitor trial progress and procedure. A respiratory physician who specialises in asthma management will provide a quality assurance review of the asthma management clinic every 6 months at both sites.

## Discussion

The clinical and cost effectiveness of an Antenatal Asthma Management Service has not been rigorously evaluated to date through a randomized controlled trial. Asthma is the most prevalent chronic medical condition to affect human pregnancy and is expected to increase in coming years, representing a significant burden for the healthcare system. This associated burden lies in coping with the increasing demands of caring for pregnant women with asthma and in dealing with its associated adverse effects on perinatal health outcomes.

The need for, and potential of, an individualized review of asthma management during pregnancy has been well demonstrated in previous research undertaken through this research group [17]. Given finite resources, however, it is unlikely that existing models of practice could cope with the increased requirements of providing an asthma management service as outlined in this protocol, highlighting the importance of this clinical and cost effectiveness analysis.

The provision of an Antenatal Asthma Management Service conducted by a respiratory nurse provides an alternative approach for the introduction of education for asthma self-management skills and the provision of an asthma action plan. Working in close consultation with each woman’s primary health care provider (i.e. GP) and hospital-based midwives and specialist physicians, the respiratory nurse is able to play a key role in guiding optimized asthma management and empowering pregnant women to manage their asthma more effectively. The introduction of this intervention has a significant potential to improve current clinical care of pregnant women with asthma and lead to a significant reduction in the incidence and severity of asthma exacerbations, improve asthma control and related quality of life and decrease the negative impact of asthma on a range of Perinatal health outcomes. These improvements in health outcomes are of major benefit to the health care system, as there are significant costs associated with managing exacerbations (i.e. unplanned medical and emergency department visits, hospitalizations), maternal complications (i.e. pre-eclampsia) and neonatal complications (i.e. low birth weight) associated with maternal asthma. In addition to immediate costs, many of these outcomes are associated with long-term morbidity for the child and adult, representing a substantial ongoing cost and health burden to society for the entire life span of these individuals [43].

The data generated from this study will provide strong evidence in support of new policies for the care of pregnant asthmatic women and models of health service delivery. A significant benefit of this proposal lies in the fact that the intervention can be introduced in any setting where pregnant women are cared for, which include hospitals, GP offices and regional health services. Furthermore, an established relationship between the research group and asthma organizations (i.e. Asthma Australia) will ensure the effective dissemination of the results and recommendations of this study to clinical practice.

## Abbreviations

ACQ: Asthma control questionnaire; FEV1: Forced expiratory volume in one second; FENO: Exhaled fraction of nitric oxide; FMC: Flinders medical centre; GP: General practitioner; ICS: Inhaled corticosteroid; IUGR: Intrauterine growth restriction; LMH: Lyell MCewin hospital; NICU: Neonatal intensive care unit; OR: Odds ratio; QALY: Quality adjusted life year; RR: Relative risk.

## Competing interests

The authors declare that they have no competing interests.

## Authors’ contributions

LEG, GD, KR, KRT, AR, BS, JB, RB, MD, JB, AW, RR, JK and VLC are all members of the AAMS study group. LEG prepared the initial draft of the AAMS protocol. The investigators (GD, KR, KRT, AR, BS, JB, RB, MD, JB, AW, RR, JK and VLC) participated in the design of the study. The AAMS study group participated in protocol development, commented on drafts of the protocol, and have read and approved the final draft of the protocol. All authors read and approved the final manuscript.

## Pre-publication history

The pre-publication history for this paper can be accessed here:

http://www.biomedcentral.com/1471-2393/14/9/prepub
